# The Impact of Foreign Trade on Health Inequality in China: Evidence From China Family Panel Studies (CFPS)

**DOI:** 10.3389/ijph.2022.1605117

**Published:** 2022-09-14

**Authors:** Pei Xu, Penghao Ye

**Affiliations:** ^1^ Business School, Yangzhou University, Yangzhou, China; ^2^ School of Economics, Hainan Open Economy Research Institute, Hainan University, Haikou, China

**Keywords:** CFPS, foreign trade, health inequality, concentration index decomposition, GMM

## Abstract

**Objectives:** To assess the health inequality caused by foreign trade in China using individual self-rated health data from China Family Panel Studies (CFPS).

**Methods:** The GMM model was used to explore the direct and indirect effects of foreign trade on health level, and the concentration index method was then used to decompose the contribution of foreign trade to health inequality.

**Results:** The direct effect of foreign trade does not contribute to the current health inequality, although the indirect effects of trade contribute to health inequality through inequalities in income and healthcare utilization. The indirect pollution effect of trade does not cause health inequality. Subsequently, the direct effect of trade aggravates the dynamic expansion trend of health inequality, whereas the indirect effects of trade alleviate the increasing trend of health inequality.

**Conclusion:** Although foreign trade improves the overall health level in China, it contributes to health inequality. Optimizing product structure of trade, adjusting income distribution, and enhancing medical securities for low-income groups are necessary to alleviate the health inequality caused by foreign trade.

## Introduction

Although health levels such as life expectancy have increased, health inequality between the rich and the poor is getting worse; this inequality is quite common in both developed and developing countries [1–4]. Based on health data including health level, total health expenditure, outpatient fee, and access to the advanced hospital from the CFPS [5], we used the concentration index (CI) method [6, 7] to calculate the income-related health inequalities indexes at the national level (Supplementary Table SA). As is shown in column (3) of Supplementary Table SA, health inequalities are very common in China with all health inequalities indicators (CIs) being significant at 1% level. Further, the mean values of health levels differ among different income groups (divided by every 25% level) over the 4 years in China (Supplementary Figure SA), indicating that pro-rich health inequality does exist in China. We further measured the health inequalities at province level over the 4 years in China and plotted the health inequalities and GDP per capita among provinces, respectively in Supplementary Figures SB, SC. Although there is some missing data for health inequality in 2009 and 2013, the results reveal that the degree of health inequality in economically underdeveloped areas (with lower GDP per capita) is generally higher than that in economically developed areas.

**FIGURE 1:**
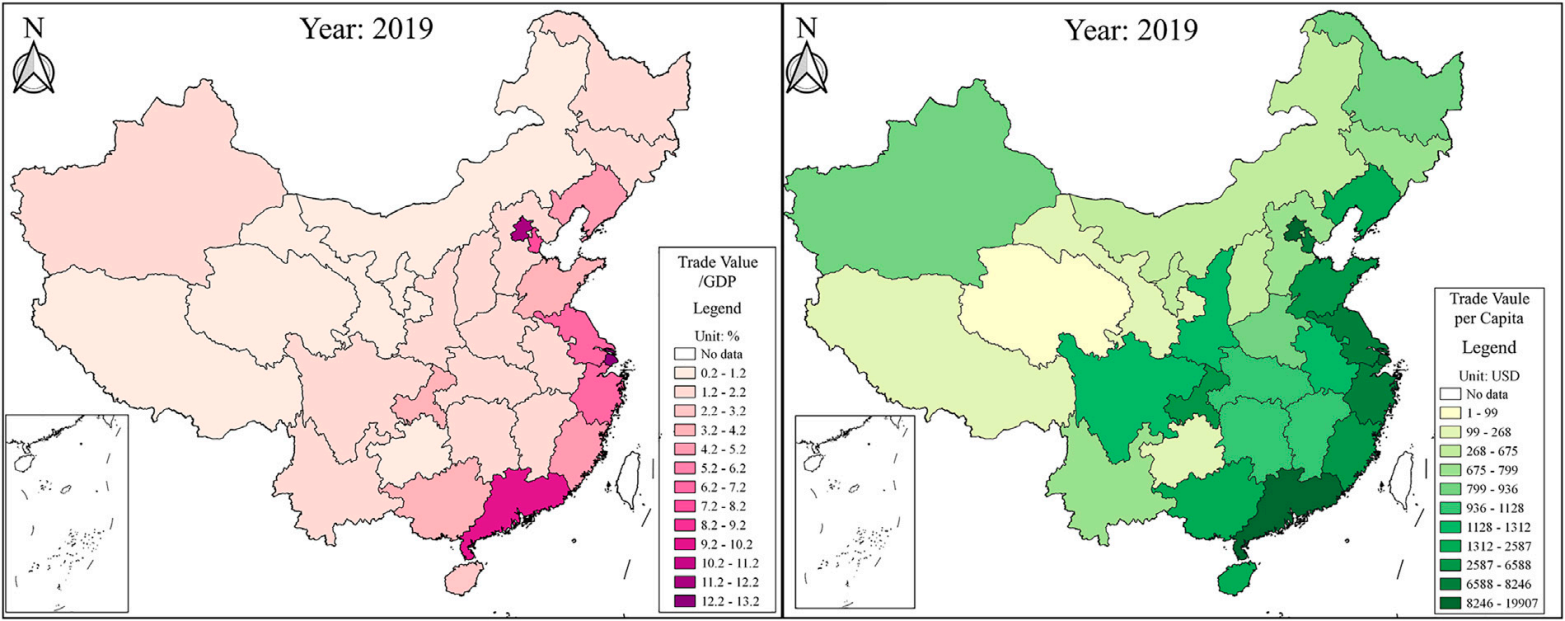
The province-level ratio of foreign trade to GDP and foreign trade per capita (China, 2019).

**FIGURE 2:**
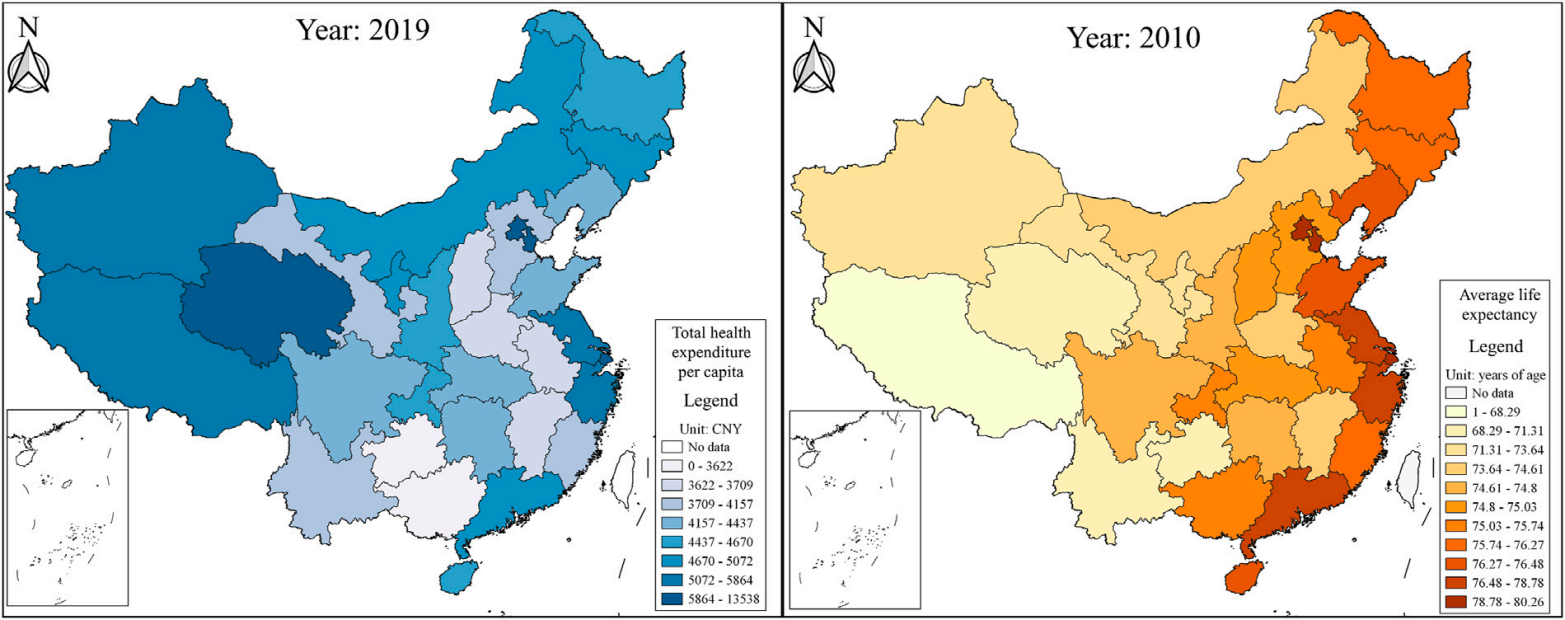
Total health expenditure per capita (China, 2019) and the average life expectancy (China, 2010).

Although research on health inequalities is becoming more prevalent over time [8–11], little attention has been paid to the interdisciplinary subject of trade and health inequality. Figure 1 shows the province-level ratio of foreign trade to GDP and foreign trade per capita in 2019 from the China Statistical Yearbook. Foreign trade-developed provinces, such as Shanghai, Beijing, Guangdong, Tianjin, Zhejiang, Jiangsu, and Fujian, are also the most economically developed coastal regions in China. Foreign trade can change the distribution of socioeconomic factors that determine socioeconomic inequalities [12, 13]. Socioeconomic inequalities are the main factors that contribute to socioeconomic inequalities [14, 15]. As such, theoretically, foreign trade may drive health inequalities. The Health China Statistical Yearbook published the latest total health expenditure per capita in 2019 but the average life expectancy was only in 2010. Figure 2 plots the two health variables. The distributions of the average life expectancy in 2010 and total health expenditure per capita in 2019 are very similar to that of the ratio of foreign trade to GDP and foreign trade per capita in 2019. Foreign trade in developed regions is linked to a higher life expectancy and more health spending, verifying that difference in foreign trade among provinces indeed contributes to health inequalities.

The health system in China has been evolving for 40 years and has now formed a basic medical insurance system that covers everyone. The health system is comprised of two different insurance systems, i.e., the basic medical insurance system for urban workers (MIUW) and the basic medical insurance system for non-working urban residents and rural residents (MINUWRR). There are three main differences between the two. Firstly, the former is mandatory, while the latter is voluntary. Secondly, the insurance fund of MIUW shall be jointly raised by the employer and the employees based on wage levels, while that of MINUWRR is mostly supported by the government, and a small part is paid by residents themselves. Thirdly, the MIUW covers both inpatient and outpatient services while the MINUWRR mainly focuses on inpatient services. An important characteristic of the health system in China is that it is governed by local authorities. That is, the health system raises more medical funds, covers more catalogs, and has a higher reimbursement proportion because of the more-advanced socioeconomic development in economically developed provinces. Foreign trade is related to the different socioeconomic development among provinces. As such, foreign trade can lead to the unequal allocation of health resources and then contribute to health inequality. The ratio of tertiary hospitals (the most advanced hospital) to all hospitals in 2019 in foreign trade developed provinces is more than three times that of the foreign trade undeveloped areas.

The specific mechanism framework of the effect of foreign trade on health inequality can be divided into direct and indirect effects (Supplementary Figure SD).

The direct effect refers to health inequalities caused by commodity consumption (i.e., food, alcohol, tobacco, etc.) in the international market. The direct contribution of foreign trade to health inequality depends on two aspects: one is the elasticity of the direct effect of foreign trade on individual health, and the other one is the inequality in foreign trade [6]. Numerous studies have shown that the elasticity is negative [16–19]. The imports and exports of unhealthy foods have led to the frequent occurrence of chronic human diseases such as obesity, diabetes, and cardiovascular diseases. China is currently experiencing a nutrition transition, resulting in dietary patterns associated with chronic disease. This dietary habit is mainly related to the increasing scales of foreign trade [19]. On the other hand, there is inequality in foreign trade among rich and poor groups, in other words, the rich participate more in international trade in processed foods, alcohol, and tobacco, narrowing the health difference between high-income and low-income groups. Therefore, we propose that the direct effect of foreign trade may not contribute to health inequalities.

The indirect effects refer to health inequality caused by foreign trade through socioeconomic inequalities (i.e., income inequality, healthcare inequality, and environmental inequality).

Income inequality is the main factor that contributes to health inequalities [14, 15, 20–22]. The rich have more capital factors, while the poor have more labor factors. Biased-capital technological progress caused by international trade contributes to income inequality by increasing the capital return ratio while decreasing the labor return ratio [23]. The rich’s willingness and ability to pay for medical insurance are much higher than the poor. Thus, foreign trade may lead to health inequalities through income inequalities. Tausch (2012) [24] found that the trade of multinational corporations led to income inequality, and the income gap was the main cause of health inequality. Chokshi (2018) [25] found that international trade exacerbated health inequalities in the United States, as high-income groups had much more access to imported medical services than low-income groups. Therefore, we propose that foreign trade may lead to health inequalities through income inequalities.

Some items of medical and health security are public goods, which need to be provided by the government. Based on the “compensation theory” put forward by Rodrik (1998) [26], the scale of government welfare expenditures such as healthcare spending is positively related to the development of foreign trade [27]. International trade in medical industries can also improve medical skills [28, 29]. As such, medical conditions are much better in regions with more developed foreign trade. According to the Health China Statistical Yearbook, the ratio of tertiary hospitals (the most advanced hospital) to all hospitals in 2019 varied greatly among provinces. The top ten are provinces with developed foreign trade, which is more than three times that of western regions with the least developed foreign trade. That is, foreign trade could contribute to inequalities in healthcare use. Inequality in healthcare utilization is another factor that contributes to health inequality [28, 30]. As such, we propose that foreign trade may lead to health inequalities through healthcare inequalities.

Environmental pollution can accelerate the depreciation of individual health [31, 32]. The rich’s willingness and ability to pay for a clean environment are higher while the poor are always exposed to areas with a high concentration of pollution, indicating that environmental inequalities could lead to health inequalities [33–36]. The effect of foreign trade on environmental pollution depends on the net effect of scale effect, structure effect, and technology effect. Technology effect tends to reduce environmental pollution while the scale effect does the opposite. Structure effect is ambiguous depending on whether international trade transfers green industrial structure [37–40]. The differences in these three effects of foreign trade among provinces can contribute to environmental inequalities [35, 41, 42]. Richardson et al. (2013) [34] found that the free trade between eastern and western counties deteriorated the environment in less-developed western counties, leading to Europe-wide mortality inequalities. Therefore, we propose that foreign trade may lead to health inequalities through environmental inequalities.

Using the health data from the CFPS from 2009–2017, this study adds a body of knowledge about effects of foreign trade on health inequalities in three aspects: 1) we differentiate the direct and indirect effects of foreign trade on health inequalities with the indirect effects focusing on income inequality, healthcare inequality, and environmental inequality; 2) we calculate the contribution proportion to health inequality by foreign trade; and 3) we further explore the changing trend of health inequality and the effect of foreign trade on the trend of inequality.

## Methods

### Data and Variables

#### Data

From 2010 to now, the CFPS has published individual or family-level data including self-rated health, age, gender, education, insurance, family size, etc. It is published every 2 years, with the latest one in 2018. We did not use the 2012 CFPS data since the values of the key variable (self-rated health) in 2012 are inconsistent with that of the other years. Besides, the statistics of CFPS are the previous year’s data, that is, the data of CFPS in 2018 is the real value of 2017. We also did not use child samples younger than 18 years old. The CFPS can only be matched with other databases at the provincial level. Other province-level data, including foreign trade, medical level, environmental pollution, industrial structure, and GDP per capita, are all from EPS China Data (EPS).

#### Variables

Health inequality ( C ) and health level ( health )*.* Referring to Fajardo-Gonzalez (2016) [43], we designed a dummy health , taking the value of one if the self-rated health is healthy or higher and zero otherwise. The health inequality is constructed through the concentration index method [6] as Eq. 1:

$$C=\frac{2}{n\mu }\overset{n}{\underset{i=1}{\sum }}{health}_{i}R_{i}-1$$ (1)

where μ is the mean of health , $R_{i}$ is the fractional rank of the i th person in the income distribution, and C is the health inequality. The bigger the value of C , the higher the level of health inequality is.

Foreign trade (*trade*). Trade dependency is an important index reflecting the development of liberalized trade [44]. Therefore, we measure *trade* by the ratio of foreign trade to GDP.

Household income per capita (*income*)*.* Referring to Deaton (2003) [45], we adopted total household income divided by family size to represent household income per capita, taken in logarithmic form.

Medical level (*medical*). Based on the EPS dataset, we selected six medical indicators: medical institutions per 10,000 people, beds per 1,000 people, health technicians per 1,000 people, assets per capita of medical and health institutions, income per capita of medical and health institutions, and out-patient visits in hospitals. Based on these six indicators, we constructed *medical* by the factor analysis method.

Environmental pollution (*pollution*). The EPS dataset provides four kinds of pollutants data: SO_2_ emissions, NH_3_-N emissions, COD emissions, and industrial wastewater emissions. Based on these four indicators, we calculated *pollution* by the factor analysis method.

Control variables*. gender* takes the value of 1 if male and 0 otherwise. *Age* is individual age. *edu* is an education dummy, taking the value of 1 with a college education or higher and 0 otherwise. *working* is the dummy, taking the value of 1 if the person is in work and 0 otherwise. *insurance* takes the value of 1 if residents are medically insured and 0 otherwise. *f_size* is total family population. *urban* takes the value of 1 for urban residents and 0 otherwise. *structure* denotes the ratio of the added value of the tertiary industry to that of secondary industry. *Pgdp* is GDP per capita in logarithmic form.

### Model Specification

We firstly designed an empirical model as Eq. 2 to analyze the possible influencing factors on health level and then decomposed the health inequality based on regression results.

$$\begin{array}{l} health_{it}=\alpha_{0}+\alpha_{1}trade_{jt}+\alpha_{2}income_{it}+\alpha_{3}medical_{jt}\\ +\alpha_{4}pollution_{jt}+\alpha_{5}trade_{jt}*\;income_{it}\\ +\alpha_{6}trade_{jt}*medical_{jt}+\alpha_{7}trade_{jt}*pollution_{jt}\\ +\alpha_{8}X_{it}+\zeta_{\mathrm{it}} \end{array}$$ (2)

All variables in Eq. 2 are the same as the section “*Variables*”. The three interactions ( ${trade}_{jt}*{income}_{it},\;{trade}_{jt}*{medical}_{jt},\;and\;{trade}_{jt}*{pollution}_{jt}$ ) are the indirect effects of foreign trade. $X_{it}$ is a vector of control variables.

## Results

### Descriptive Statistics

Supplementary Table SB displays the descriptive statistics of the main variables. Health dummy (*health*) averaged 0.734 with a standard deviation of 0.442, indicating health level differing among residents. The mean value, standard deviation, and maximum value of *trade* are 0.048, 0.055, and 0.202, respectively but the minimum value is only 0.007, indicating that there are great differences in foreign trade among provinces.

### Static Result

#### Baseline Model

Table 1 shows the baseline results. Since the health level is a dummy variable, the Probit model is used and the OLS model is used for comparative analysis. Considering the possible endogenous problems, the GMM model used the lag period of the core explanatory variable as its own instrumental variable to avoid endogeneity [46].

**TABLE 1:** Regression results (China, 2009, 2013, 2015, and 2017).

	Probit	OLS	GMM	Probit	OLS	GMM
	(1)	(2)	(3)	(4)	(5)	(6)
*trade*	−10.115***	−2.827***	−5.353***	−11.227***	−3.154***	−5.352***
	(1.87)	(0.61)	(1.58)	(1.87)	(0.61)	(1.58)
trade*income	0.444**	0.134**	0.304*	0.509***	0.154***	0.302*
	(0.17)	(0.06)	(0.16)	(0.17)	(0.06)	(0.16)
trade*medical	3.008***	0.830***	1.186***	3.174***	0.878***	1.186***
	(0.42)	(0.14)	(0.21)	(0.42)	(0.14)	(0.21)
trade*pollution	0.983***	0.245***	0.389***	0.992***	0.241***	0.392***
	(0.22)	(0.07)	(0.13)	(0.22)	(0.07)	(0.13)
*income*	0.111***	0.032***	0.078***	0.111***	0.032***	0.078***
	(0.01)	(0.00)	(0.01)	(0.01)	(0.00)	(0.01)
*medical*	0.360***	0.106***	0.162***	0.390***	0.117***	0.161***
	(0.04)	(0.01)	(0.02)	(0.04)	(0.01)	(0.02)
*pollution*	−0.049***	−0.013***	−0.015**	−0.035**	−0.008	−0.015**
	(0.02)	(0.01)	(0.01)	(0.02)	(0.01)	(0.01)
*gender*	0.230***	0.075***	0.074***	0.230***	0.075***	0.074***
	(0.01)	(0.00)	(0.00)	(0.01)	(0.00)	(0.00)
*f_size*	0.011***	0.004***	0.009***	0.011***	0.004***	0.009***
	(0.00)	(0.00)	(0.00)	(0.00)	(0.00)	(0.00)
*age*	−0.024***	−0.008***	−0.007***	−0.023***	−0.008***	−0.007***
	(0.00)	(0.00)	(0.00)	(0.00)	(0.00)	(0.00)
*edu*	0.188***	0.037***	0.015	0.172***	0.032***	0.015
	(0.03)	(0.01)	(0.01)	(0.03)	(0.01)	(0.01)
*working*	0.187***	0.066***	0.087***	0.196***	0.069***	0.087***
	(0.02)	(0.01)	(0.01)	(0.02)	(0.01)	(0.01)
*structure*	0.085**	0.025**	0.055***	0.129***	0.042***	0.055***
	(0.04)	(0.01)	(0.01)	(0.04)	(0.01)	(0.01)
*pgdp*	0.437***	0.119***	0.182***	0.493***	0.140***	0.182***
	(0.05)	(0.02)	(0.02)	(0.05)	(0.02)	(0.02)
*insurance*	0.005	0.006	−0.002	0.010	0.007	−0.002
	(0.02)	(0.01)	(0.01)	(0.02)	(0.01)	(0.01)
*urban*	0.036**	0.015***	−0.004	0.035**	0.014***	−0.004
	(0.01)	(0.01)	(0.01)	(0.01)	(0.00)	(0.01)
*province*	−0.001**	−0.001***	−0.000	−0.001	−0.000*	−0.000
	(0.00)	(0.00)	(0.00)	(0.00)	(0.00)	(0.00)
Time FE	No	No	No	Yes	Yes	Yes
Pseudo R^2^/R^2^	0.094	0.113	0.101	0.095	0.115	0.101
N	43,882	43,882	36,701	43,882	43,882	36,701

Note: ***, **, and * represent the estimated coefficient is statistically significant at the 1%, 5%, and 10% levels respectively. The values in the brackets are the Standard errors.

Columns (1)–(6) of Table 1 all reveal that the direct effect of foreign trade is significantly negative on the health level, while the indirect effects of foreign trade significantly improve the health level by increasing household income per capita, improving the medical level, and reducing the environmental pollution.

Using four kinds of health data, i.e., health level (*health*), total health expenditure [*ln*(*metotal*)], outpatient fee [*ln*(*outpafee*)], and access to the advanced hospitals (*meaccess*) in 2015 and 2017 from CFPS, we constructed the Structural Equation Modelling (SEM) to conduct the robustness test (Supplementary Table SC). In columns (1)–(4), the coefficients of *trade* to these four health variables are all significantly negative, indicating the negative direct effect of trade on residents’ health. For the indirect effects, the coefficients of *trade* to *income* are all significantly positive and the coefficients of *income* to three health variables [*health*, *ln*(*outpafee*), and *meaccess*] are also significantly positive, indicating that foreign trade can improve individual health level by increasing household income per capita. The coefficients of *trade* to *medical* are all significantly positive and the coefficients of *medial* to four health variables are all significantly positive, indicating that foreign trade can improve individual health level by improving medical level. The coefficients of *trade* to *pollution* are all significantly negative and the coefficients of *pollution* to four health variables are all significantly negative, indicating that foreign trade can improve individual health level by reducing environmental pollution. In sum, the results of SEM are consistent with baseline findings in Table 1.

#### Decomposition Results

Using concentration index method [6], we then decomposed the health inequality based on baseline results as Eq. 3.

$$C=\underset{k}{\sum }\left(\beta_{k}{\bar{x}}_{k}/u\right)C_{k}+GC_{\varepsilon }/u$$ (3)

where u is the mean of health , ${\bar{x}}_{k}$ is the mean of $x_{k}$ , and $C_{k}$ is the concentration index for $x_{k}$ (defined analogously to C ). $GC_{\varepsilon }$ is a generalized concentration index for $\varepsilon_{i}$ .

Table 2 reports the decomposition results. Columns (1)–(5) represent the regression coefficients, mean, elasticity, concentration indices, and contributions to C . Especially, Contribution to C=Elasticity×Concentration index . The health inequality ( C) in the last line is 0.074, which means there exists a pro-rich health inequality. Column (5) makes it clear that the bulk of inequality in health was caused by inequalities in foreign trade (*trade*), household income per capita (*income*), medical level (*medical*), age (*age*), and GDP per capita (*pgdp*). In this paper, we mainly focus on the effect of foreign trade on health inequality.

**TABLE 2:** Inequality decompositions (China, 2009, 2013, 2015, and 2017).

Variables	Coefficients	Mean	Elasticity	Concentration Indices	Contributions to C
	(1)	(2)	(3)	(4)	(5)
*trade*	−5.352	0.043	−0.342	0.186***	−0.064
trade*income	0.302	0.421	0.189	0.256***	0.048
trade*medical	1.186	0.060	0.106	0.047***	0.005
trade*pollution	0.392	0.037	0.022	0.0006	0.000
*income*	0.078	9.498	1.100	0.155***	0.171
*medical*	0.161	0.384	0.092	0.181***	0.017
*pollution*	−0.015	0.377	−0.008	0.0005	−0.000
*gender*	0.074	0.505	0.055	0.024***	0.001
*f_size*	0.009	4.221	0.056	−0.092***	−0.005
*age*	−0.007	48.188	−0.501	−0.027***	0.014
*edu*	0.015	0.068	0.002	0.554***	0.001
*working*	0.087	0.750	0.097	0.071***	0.007
*structure*	0.055	1.175	0.096	0.088***	0.008
*pgdp*	0.182	10.754	2.906	0.166***	0.482
*insurance*	−0.002	0.922	−0.003	−0.024***	0.000
*urban*	−0.004	0.510	−0.003	0.387***	−0.001
C	0.074***				

Note: ***, **, and * represent the estimated coefficient is statistically significant at the 1%, 5%, and 10% levels, respectively.

The next step is to distinguish between the direct and indirect effects of foreign trade on health inequalities. For the direct contribution, the elasticity coefficient of *trade* is negative (−0.342), indicating that the direct effect of foreign trade on the health level is negative. The concentration index of *trade* is positive (0.186), which is probably because the rich participate in more international consumption of goods high in sugar, fat, and salt, tobacco, and alcohol, bearing more health risks related to foreign trade. For example, richer consumers who buy COVID-19-contaminated imported cherries are at higher health risks than those lower-income groups. Therefore, the direct effect of foreign trade does not contribute to the current health inequality by increasing the health risks bore by high-income groups.

Among the three indirect contributions, the trade * income is the biggest, the trade * medical the second, and the trade * pollution the least. Specifically, first, the concentration index of trade *  income is positive (0.256), indicating that the income mediating effect of foreign trade further exacerbates income inequality. China’s partial capital technology progress caused by foreign trade makes the ratio of return on capital and technology further increase, while the ratio of return on labor factor decreases [47]. Therefore, foreign trade further enlarges the income gap and in turn contributes to income-related health inequality.

Second, the elasticity and concentration index of the interaction term trade * medical are both significantly positive (0.106 and 0.047), indicating that although foreign trade improves the health level by promoting medical skills, it also leads to health inequality through medical inequality. The inequality in trade *  medical means that the benefits of foreign trade in improving medical skills are mostly enjoyed by high-income groups. High-income groups can afford advanced imported medicines and medical facilities while the poor have much less access to advanced medical treatments. Therefore, foreign trade contributes to health inequalities through inequality in healthcare use.

Finally, the elasticity of trade * pollution is positive (0.022), that is, foreign trade can improve residents’ health by reducing environmental pollution. The concentration index is positive but not significant, indicating foreign trade does not lead to environmental inequality among provinces. In foreign trade-undeveloped provinces, foreign trade will not cause serious environmental pollution since the scale of foreign trade is small in these areas. In foreign trade-developed provinces, although the scale effect of foreign trade tends to deteriorate the environment, the structure effect and technology effect are likely to alleviate environmental pollution [48]. The industrial structure of foreign trade has been becoming cleaner, and the technology of foreign trade has been improving with the green economic transformation in China in recent years and the higher environmental requirements in international markets. Foreign trade does not cause environmental inequality among provinces. Therefore, foreign trade does not contribute to health inequality through environmental inequality.

### Dynamic Results

The column headed with “change” in Table 3 indicates that the bulk of the change of health inequality between 2015 and 2017 was due to changes in respect of the direct and indirect effects of foreign trade. The net change of C was 0.021, that is, the health inequality is widening. The net change of *trade* is 0.053, indicating that the direct effect contributes to the dynamic expansion of health inequalities. The change of concentration index of *trade* dropped slightly (i.e., from 0.216 to 0.200) but was still significantly positive, which means the distribution of this exchange is still mainly in high-income groups. The elasticity of *trade* changed from −0.508 to −0.284, that is, the negative elasticity of foreign trade with respect to health significantly decreased. Therefore, the direct effect of foreign trade boosted the growing trend of health inequality on the whole.

**TABLE 3:** The change of health inequality (China, 2015 and 2017).

	Coefficients		Means		Elasticities		Concentration indices		Contributions to C		
	2015	2017	2015	2017	2015	2017	2015	2017	2015	2017	Change
*trade*	−7.596	−5.004	0.044	0.040	−0.508	−0.284	0.216	0.200	−0.110	−0.057	0.053
trade*income	0.416	0.323	0.426	0.392	0.269	0.180	0.299	0.284	0.081	0.051	−0.030
trade*medical	1.496	1.069	0.052	0.066	0.118	0.100	0.045	0.046	0.005	0.005	−0.001
trade*pollution	0.575	0.410	0.058	0.012	0.051	0.007	0.005	−0.003	0.000	−0.000	−0.000
*health*			0.658	0.705			0.137	0.158			0.021

By contrast, the change of trade * income tends to narrow the dynamic trend of health inequality with the change in equality being −0.03. The decreasing concentration index of trade * income indicates that the income inequality caused by foreign trade has been somewhat alleviated. Although the pro-capital technological progress of foreign trade led to an income gap, foreign trade also enlarged the market demand and supply which increases the income of low-income groups. In addition, the competitive effect of foreign trade can reduce monopoly profits and commodity prices, raising the relative income of low-income groups. Moreover, the elasticity of trade * income decreased from 0.299 to 0.284, indicating that the sensitivity of health to the income mediating effect of foreign trade slightly decreased. Therefore, foreign trade tends to generally mitigate the expansion of health inequality through the mediating income effect.

The change of trade * medical also tends to narrow the health inequality with the change being −0.001. The increase of the concentration index of trade * medical indicates that the inequality in medical level caused by foreign trade has slightly increased. Since the rich can afford costly imported medicines and medical equipment, the improvement of medical level by foreign trade is more beneficial to the rich. But the elasticity coefficient of the interaction item trade * medical declined from 0.118 to 0.1, indicating that the promoting effect of foreign trade on the health level by improving the medical level has been weakened. On the whole, foreign trade tends to alleviate the expansion trend of health inequality through the mediating effect of the medical level.

The change of trade * pollution to health inequality is negligible, that is, the effect of foreign trade on health inequality through mediating environmental pollution is insignificant.

We further used the Oaxaca decomposition method [49] as Eq. 4 to pinpoint to what extent these changes were due to changes in elasticities rather than changes in inequality.

∆C=∑kηkt−1(Ckt−Ckt−1)+∑kCkt(ηkt−ηkt−1)+Δ(GCεtμt) (4)

where $\eta_{kt}$ is denoted as the elasticity of health with respect to $x_{k}$ at time t ; other variables are in keeping with Eq. 3.

The Oaxaca decomposition results are shown in Table 4. The net change of inequalities ( ∆C ) in the direct effect of foreign trade (*trade*) was significantly positive. By contrast, changes in the indirect effects of foreign trade (trade * income and trade * medical) were in opposite directions. The change in respect of the direct effect of foreign trade makes for more inequality in health inequality. For indicators (*trade*, trade * income, and trade * medical), it is the changing elasticity—rather than changing inequality—that accounts for the bulk of the changes in health equalities.

**TABLE 4:** Oaxaca-type decomposition for change in inequality (China, 2015 and 2017).

	Variables	∆C	η∆C	C∆η
2015–2017	*trade*	0.053	0.008	0.045
	trade*income	−0.030	−0.004	−0.025
	trade*medical	−0.001	0.000	−0.001
	trade*pollution	−0.000	0.000	−0.000

## Discussion

We explored health inequality from the perspective of foreign trade, and found intriguing results. First, health inequality is expanding at the present stage, and foreign trade is one of the main causes of health inequality. Second, the direct and indirect effects of foreign trade contribute differently to health inequality. Specifically, the direct effect of foreign trade does not contribute to current health inequality, while the indirect effects of foreign trade contribute to health inequality through mediating income inequality and healthcare inequality but the indirect pollution effect of trade is negligible. Specifically, among indirect effects, the contribution of income is much larger than the medical effect. Lastly, in the dynamic expansion trend of health inequality, the direct effect of foreign trade tends to increase its expansion trend, while the indirect effects of foreign trade can ease the expansion trend of health inequality. Based on empirical results, we suggest that: Firstly, although the direct effect of trade does not contribute to health inequality, it increases the health risks of the rich. Thus, increasing the import tax on unhealthy food to reduce the trade-related health risk is recommended. Secondly, governments can implement foreign trade policies to alleviate the income inequality related to foreign trade. Lastly, the government can facilitate international medical assistance cooperation and distribute medical resources according to residents’ health needs to narrow the inequalities in healthcare utilization.

## References

[1] David B Alex B Durka D. A Vision for Population Health: Towards a Healthier Future (2018).

[2] Li Z Kim R Subramanian SV. Economic-related Inequalities in Child Health Interventions: An Analysis of 65 Low- and Middle-Income Countries. Soc Sci Med (2021) 277:113816. 10.1016/j.socscimed.2021.113816 33848717

[3] Murphy KA Ellison-Barnes A Johnson EN Cooper LA. The Clinical Examination and Socially At-Risk Populations: The Examination Matters for Health Disparities. Med Clin North Am (2018) 102:521–32. 10.1016/j.mcna.2017.12.013 29650073

[4] Morton A. Aversion to Health Inequalities in Healthcare Prioritisation: a Multicriteria Optimisation Perspective. J Health Econ (2014) 36:164–73. 10.1016/j.jhealeco.2014.04.005 24831800

[5] China Family Panel Studies. CFPS. Available at: https://opendata.pku.edu.cn/dataverse/CFPS (Accessed April 13, 2021).

[6] Wagstaff A Van Doorslaer E Watanabe N. Ondecomposingthecausesofhealthsector Inequalities with an Application to Malnutrition Inequalities in Vietnam. J Econom (2003) 112:207–23. 10.1016/s0304-4076(02)00161-6

[7] Bleichrodt H Rohde KI Van Ourti T. An Experimental Test of the Concentration index. J Health Econ (2012) 31:86–98. 10.1016/j.jhealeco.2011.12.003 22307035 PMC4753067

[8] Rathmann K Pfortner TK Hurrelmann K Osorio AM Bosakova L Elgar FJ The Great Recession, Youth Unemployment and Inequalities in Psychological Health Complaints in Adolescents: a Multilevel Study in 31 Countries. Int J Public Health (2016) 61:809–19. 10.1007/s00038-016-0866-0 27502510

[9] Liu K Cook B Lu C. Health Inequality and Community-Based Health Insurance: a Case Study of Rural Rwanda with Repeated Cross-Sectional Data. Int J Public Health (2019) 64:7–14. 10.1007/s00038-018-1115-5 29947821

[10] Smith KE Macintyre AK Weakley S Hill SE Escobar O Fergie G. Public Understandings of Potential Policy Responses to Health Inequalities: Evidence from a UK National Survey and Citizens' Juries in Three UK Cities. Soc Sci Med (2021) 291:114458. 10.1016/j.socscimed.2021.114458 34655938 PMC8711040

[11] Nie P Clark AE D'Ambrosio C Ding L. Income-related Health Inequality in Urban China (1991-2015): The Role of Homeownership and Housing Conditions. Health Place (2022) 73:102743. 10.1016/j.healthplace.2022.102743 35045352

[12] Erreygers G Van Ourti T. Measuring Socioeconomic Inequality in Health, Health Care and Health Financing by Means of Rank-dependent Indices: a Recipe for Good Practice. J Health Econ (2011) 30:685–94. 10.1016/j.jhealeco.2011.04.004 21683462 PMC3158909

[13] Greaney TM Karacaovali B. Editorial: Trade, Growth and Economic Inequality in the Asia-Pacific Region. J Asian Econ (2017) 48:1–5. 10.1016/j.asieco.2016.12.001

[14] Coveney M Garcia-Gomez P van Doorslaer E Van Ourti T. Thank Goodness for Stickiness: Unravelling the Evolution of Income-Related Health Inequalities before and after the Great Recession in Europe. J Health Econ (2020) 70:102259. 10.1016/j.jhealeco.2019.102259 31931267

[15] Hurley J Mentzakis E Walli-Attaei M. Inequality Aversion in Income, Health, and Income-Related Health. J Health Econ (2020) 70:102276. 10.1016/j.jhealeco.2019.102276 31955864

[16] An R Guan C Liu J Chen N Clarke C. Trade Openness and the Obesity Epidemic: a Cross-National Study of 175 Countries during 1975–2016. Ann Epidemiol (2019) 37:31–6. 10.1016/j.annepidem.2019.07.002 31399309

[17] Jensen HT Keogh-Brown MR Shankar B Aekplakorn W Basu S Cuevas S International Trade, Dietary Change, and Cardiovascular Disease Health Outcomes: Import Tariff Reform Using an Integrated Macroeconomic, Environmental and Health Modelling Framework for Thailand. SSM Popul Health (2019) 9:100435. 10.1016/j.ssmph.2019.100435 31649995 PMC6804685

[18] Kusche H Hanel R. Consumers of Mislabeled Tropical Fish Exhibit Increased Risks of Ciguatera Intoxication: A Report on Substitution Patterns in Fish Imported at Frankfurt Airport, Germany. Food Control (2021) 121:107647. 10.1016/j.foodcont.2020.107647

[19] Thow AM. Trade Liberalisation and the Nutrition Transition: Mapping the Pathways for Public Health Nutritionists. Public Health Nutr (2009) 12:2150–8. 10.1017/S1368980009005680 19433005

[20] Wildman J. Income Related Inequalities in Mental Health in Great Britain: Analysing the Causes of Health Inequality over Time. J Health Econ (2003) 22:295–312. 10.1016/s0167-6296(02)00101-7 12606147

[21] Allanson P Gerdtham UG Petrie D. Longitudinal Analysis of Income-Related Health Inequality. J Health Econ (2010) 29:78–86. 10.1016/j.jhealeco.2009.10.005 19954852

[22] Chatterji P Lahiri K Song J. The Dynamics of Income-Related Health Inequality Among American Children. Health Econ (2013) 22:623–9. 10.1002/hec.2823 22514158 PMC4240021

[23] Acemoglu E. Directed Technical Change. Rev Econ Stud (2002) 69:781–809. 10.1111/1467-937x.00226

[24] Tausch A. A Globalization-Oriented Perspective on Health, Inequality and Socio-Economic Development. Int J Health Plann Manage (2012) 27:2–33. 10.1002/hpm.1090 22383449

[25] Chokshi DA. Income, Poverty, and Health Inequality. Jama (2018) 319:1312–3. 10.1001/jama.2018.2521 29614168

[26] Rodrik D. Why Do More Open Economies Have Bigger Governments? J Polit Economy (1998) 106:997–1032. 10.1086/250038

[27] Waitzkin H Jasso-Aguilar R Landwehr A Mountain C. Global Trade, Public Health, and Health Services: Stakeholders' Constructions of the Key Issues. Soc Sci Med (2005) 61:893–906. 10.1016/j.socscimed.2005.01.010 15955394

[28] Bohme SR. Two Opportunities to Improve Public Health through US Trade Policy. Int J Occup Environ Health (2010) 16:357–9. 10.1179/107735210799160129 20662428

[29] Papageorgiou C Savvides A Zachariadis M. International Medical Technology Diffusion. J Int Econ (2007) 72:409–27. 10.1016/j.jinteco.2006.09.008

[30] McNamara C. Trade Liberalization and Social Determinants of Health: A State of the Literature Review. Soc Sci Med (2017) 176:1–13. 10.1016/j.socscimed.2016.12.017 28110222

[31] Grossman M. On the Concept of Health Capital and the Demand for Health. J Polit Econ (1972) 80:223–55. 10.1086/259880

[32] Coneus K Spiess CK. Pollution Exposure and Child Health:Evidence for Infants and Toddlers in Germany. J Health Econ (2012) 31:180–96. 10.1016/j.jhealeco.2011.09.006 22030091

[33] Pierangeli I Nieuwenhuijsen MJ Cirach M Rojas-RueDa D. Health Equity and burden of Childhood Asthma - Related to Air Pollution in Barcelona. Environ Res (2020) 186:109067. 10.1016/j.envres.2019.109067 32037015

[34] Richardson EA Pearce J Tunstall H Mitchell R Shortt NK. Particulate Air Pollution and Health Inequalities: A Europe-wide Ecological Analysis. Int J Health Geogr (2013) 12:34–7. 10.1186/1476-072X-12-34 23866049 PMC3720269

[35] Bontems P Gozlan E. Trade, Environment, and Income Inequality: An Optimal Taxation Approach. J Public Econ Theor (2018) 20:557–81. 10.1111/jpet.12288

[36] Samoli E Stergiopoulou A Santana P Mitsakou C Dimitroulopoulou C Bauwelinck M Spatial Variability in Air Pollution Exposure in Relation to Socioeconomic Indicators in Nine European Metropolitan Areas: A Study on Environmental Inequality. Environ Pollut (2019) 249:345–53. 10.1016/j.envpol.2019.03.050 30909127

[37] Grossman GM Krueger AB. Environmental Impacts of a North American Free Trade Agreement. Cambridge, MA: NBER (1991). p. 3914. 10.3386/w3914

[38] Antweiler W Copeland BR Tm S. Is Free Trade Good for the Environment. Am Econ Rev (2001) 91:877–908. 10.1257/aer.91.4.877

[39] Liu M Zhang B Liao X. Women with Polycystic Ovary Syndrome (PCOS) Have Reduced Melatonin Concentrations in Their Follicles and Have Mild Sleep Disturbances. BMC Womens Health (2022) 22:79. 10.1186/s12905-022-01661-w 35313872 PMC8935689

[40] Duan Y Yan B. Economic Gains and Environmental Losses from International Trade: A Decomposition of Pollution Intensity in China's Value-Added Trade. Energ Econ (2019) 83:540–54. 10.1016/j.eneco.2019.08.002

[41] Chen W Kang JN Han MS. Global Environmental Inequality: Evidence from Embodied Land and Virtual Water Trade. Sci Total Environ (2021) 783:146992. 10.1016/j.scitotenv.2021.146992 33865121

[42] Wang Y Xiong S Ma X. Carbon Inequality in Global Trade: Evidence from the Mismatch between Embodied Carbon Emissions and Value Added. Ecol Econ (2022) 107398. 10.1016/j.ecolecon.2022.107398

[43] Fajardo-Gonzalez J. Inequality of Opportunity in Adult Health in Colombia. J Econ Inequal (2016) 14:395–416. 10.1007/s10888-016-9338-2

[44] Ma T Wang Y. Globalization and Environment: Effects of International Trade on Emission Intensity Reduction of Pollutants Causing Global and Local Concerns. J Environ Manage (2021) 297:113249. 10.1016/j.jenvman.2021.113249 34284328

[45] Deaton A. Health, Income, and Inequality. (Research Summaries). Cambridge, MA: Nber Reporter (2003).

[46] Kong W Yang K. Efficient GMM Estimation of a Spatial Autoregressive Model with an Endogenous Spatial Weights Matrix. Econ Lett (2021) 110090. 10.1016/j.econlet.2021.110090

[47] Jung S Lee J-D Hwang W-S Yeo Y. Growth versus Equity: A CGE Analysis for Effects of Factor-Biased Technical Progress on Economic Growth and Employment. Econ Model (2017) 60:424–38. 10.1016/j.econmod.2016.10.014

[48] Zhang S Collins AR Etienne XL Ding R. The Environmental Effects of International Trade in China: Measuring the Mediating Effects of Technology Spillovers of Import Trade on Industrial Air Pollution. Sustainability (2021) 6895. 10.3390/su13126895

[49] Oaxaca RL. Male-Female Wage Differentials in Urban Labor Markets. Int Econ Rev (1973) 14:693–709. 10.2307/2525981

