# Supplementary material for: The Impact of Foreign Trade on Health Inequality in China: Evidence From China Family Panel Studies (CFPS)
Source: Int J Public Health. 2022 Sep 14;67:1605117. doi: 10.3389/ijph.2022.1605117 (PMC9515319; doi:10.3389/ijph.2022.1605117)
Supplement: Supplementary file 2 [file DataSheet1.docx]

**International Journal of Public Health**

**The Impact of Foreign Trade on Health Inequality in China: Evidence from China Family Panel Studies (CFPS)**

Pei Xu,^1^ Penghao Ye,^2,*^

^1^Business School, Yangzhou University, Yangzhou 225009, China; xp007892@yzu.edu.cn

^2^ School of Economics, Hainan Open Economy Research Institute, Hainan University, Haikou 570228, China; paulyph@hainau.edu.cn

* **Correspondence**: Penghao Ye

Address: No.58 Renmin Avenue, Hainan University, Haikou 570228, China.

Email: [paulyph@hainanu.edu.cn](mailto:paulyph@hainanu.edu.cn)

**Supplementary material**

**Table A** Income-related health inequalities (China, 2009,2013, 2015, and 2017)

| Index | year | No. | CI | S.D. | P-value |
| --- | --- | --- | --- | --- | --- |
|  | (1) | (2) | (3) | (4) | (5) |
| *health level* | 2009 | 30,826 | 0.259*** | 0.010 | 0.0000 |
|  | 2013 | 33,363 | 0.122*** | 0.007 | 0.0000 |
|  | 2015 | 35,238 | 0.137*** | 0.006 | 0.0000 |
|  | 2017 | 29,552 | 0.158*** | 0.007 | 0.0000 |
| *total health expenditure* | 2015 | 31,919 | 0.060*** | 0.013 | 0.0000 |
|  | 2017 | 29,191 | 0.051*** | 0.014 | 0.0003 |
| *outpatient fee* | 2015 | 31,708 | 0.071*** | 0.010 | 0.0000 |
|  | 2017 | 28,880 | 0.064*** | 0.015 | 0.0000 |
| *access to hospital* | 2015 | 31,844 | 0.221*** | 0.005 | 0.0000 |
|  | 2017 | 29,068 | 0.205*** | 0.005 | 0.0000 |

**Table B** Descriptive statistics (China, 2009,2013, 2015, and 2017)

| Variables | N | Mean | S. D. | Min. | Max. |
| --- | --- | --- | --- | --- | --- |
| *health* | 134,606 | 0.734 | 0.442 | 0 | 1 |
| *trade* | 132,434 | 0.048 | 0.055 | 0.007 | 0.202 |
| *income* | 129,002 | 9.175 | 1.118 | 0.474 | 15.241 |
| *medical* | 134,633 | -0.048 | 0.988 | -3.794 | 1.354 |
| *pollution* | 134,633 | 0.344 | 0.986 | -1.299 | 2.671 |
| *gender* | 134,618 | 0.495 | 0.500 | 0 | 1 |
| *f_size* | 133,316 | 4.294 | 1.984 | 1 | 26 |
| *age* | 128,776 | 46.790 | 16.693 | 18 | 110 |
| *edu* | 89,516 | 0.061 | 0.239 | 0 | 1 |
| *working* | 122,875 | 0.686 | 0.464 | 0 | 1 |
| *structure* | 134,633 | 1.054 | 0.424 | 0.518 | 4.237 |
| *pgdp* | 134,633 | 10.566 | 0.502 | 9.241 | 11.651 |
| *insurance* | 100,042 | 0.912 | 0.283 | 0 | 1 |
| *urban* | 131,112 | 0.483 | 0.500 | 0 | 1 |

**Table C** Regression results of SEM model (China, 2009,2013, 2015, and 2017)

|  | *health* | *ln(metotal)* | *ln(outpafee)* | *meccess* |
| --- | --- | --- | --- | --- |
|  | (1) | (2) | (3) | (4) |
| *trade* | -0.914*** | -3.924*** | -5.858*** | -5.441*** |
|  | (0.12) | (0.89) | (0.86) | (0.40) |
| *income* | 0.045*** | 0.028 | 0.038** | 0.237*** |
|  | (0.00) | (0.02) | (0.02) | (0.01) |
| *medical* | 0.034*** | 0.255*** | 0.190*** | 0.278*** |
|  | (0.01) | (0.06) | (0.06) | (0.03) |
| *pollution* | -0.009** | -0.046* | -0.086*** | -0.023* |
|  | (0.00) | (0.03) | (0.03) | (0.01) |
| *income<-*  *trade* | 6.227*** | 6.236*** | 6.250*** | 6.238*** |
|  | (0.09) | (0.09) | (0.09) | (0.09) |
| *medical<-*  *trade* | 17.314*** | 17.268*** | 17.297*** | 17.319*** |
|  | (0.05) | (0.05) | (0.05) | (0.05) |
| *pollution<-*  *trade* | -2.109*** | -2.184*** | -2.125*** | -2.093*** |
|  | (0.10) | (0.10) | (0.10) | (0.10) |
| N | 44,023 | 43,556 | 43,697 | 43,901 |

Note: ***, **, and * represent the estimated coefficient is statistically significant at the 1%, 5%, and 10% levels respectively. The values in the brackets are the Standard errors.

**Figure A** The mean values of health level among different income groups (China, 2009,2013, 2015, and 2017)


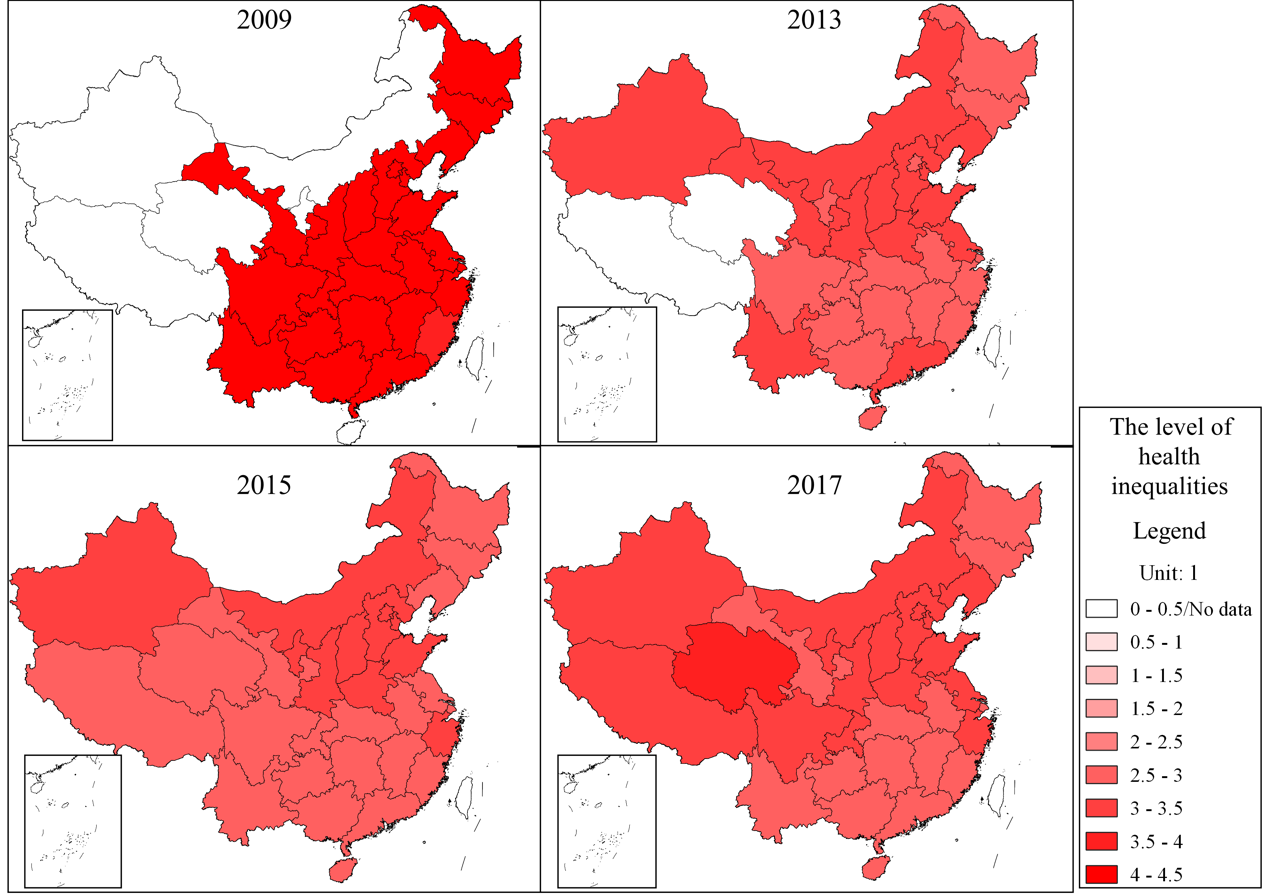


**Figure B** The level of health inequalities among provinces (China, 2009,2013, 2015, and 2017)


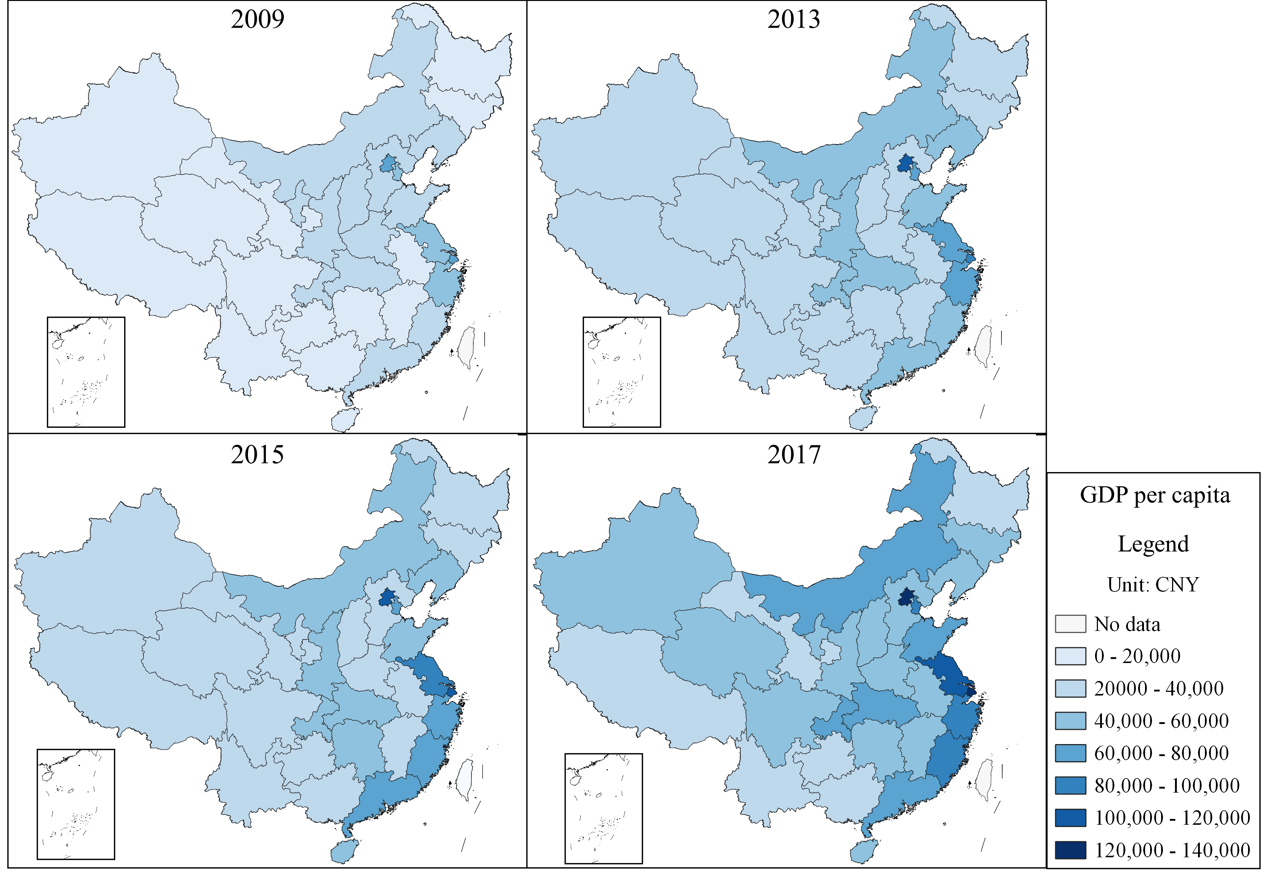


**Figure C** The GDP per capita among provinces (China, 2009,2013, 2015, and 2017)


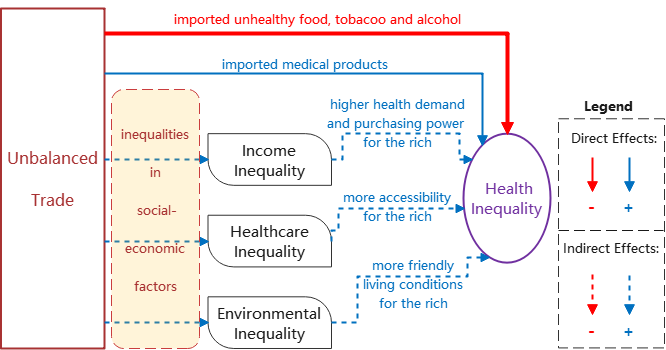


**Figure D** The transmitting mechanism of direct and indirect effects to health inequality (Source: author’s self-made)
